# 

**Published:** 1830-09

**Authors:** 


### MONTHLY LIST OF MEDICAL BOOKS.

Mémorial des Hopitaux du Midi, et de la Clinique de Montpellier; par le Prof. DELPECH. Juin 1830, 2e Année. No. 18, tome 2. Montpellier.

Practical Remarks on the Nature and Effects of the expressed Oil of the Croton Tiglium, with Cases illustrative of its Efficacy in the Cure of various Diseases. By MICHAEL J. SHORT, M.D. Plate. 8vo. pp. 63. London, 1830.

Appendix to the Second Edition of a Series of Observations on Strictures, &c. By R. A. STAFFORD. 8vo. pp. 52; stitched.

A Treatise on Pulmonary Consumption, its Prevention and Remedy. By JOHN MURRAY, F.S.A. F.L.S. F.H.S. F.G.S. &c. 8vo. pp. 156.

The Family Library, No. XIV. Lives of British Physicians. 12mo. pp. 341. Murray, 1830.

Practical Observations on Leucorrhœa, Fluor Albus, or "Weakness;" with Cases illustrative of a new Mode of Treatment. By GEORGE JEWEL, Member of the Royal College of Surgeons, Lecturer on Midwifery, &c. 8vo. pp. 108. Wilson, London, 1830.

A Treatise on the Minute Anatomy of the Bones. By ANTONIO SCARPA, Professor of Clinical Surgery in the University of Pavia. Translated from the last Latin Edition. 16mo. pp. 70. London: Bulcock, Chelsea, 1830.

### NOTICES.

*Communications received from* Dr. and Mr. D. GRIFFIN, Dr. ALEX. THOMSON, Mr. GILBERT BURNETT, Mr. GUTHRIE, Mr. JENNINGS, *of Leamington Spa,* Mr. HOBART, Dr. DOBSON, *and* Mr. WILLIAMS.

